# Vascular basement membranes as pathways for the passage of fluid into and out of the brain

**DOI:** 10.1007/s00401-016-1555-z

**Published:** 2016-03-14

**Authors:** Alan W. J. Morris, Matthew MacGregor Sharp, Nazira J. Albargothy, Rute Fernandes, Cheryl A. Hawkes, Ajay Verma, Roy O. Weller, Roxana O. Carare

**Affiliations:** Faculty of Medicine, University of Southampton, Southampton General Hospital, MP806, Tremona Road, Southampton, Hampshire SO16 6YD UK; Biogen, Binney Street, Cambridge, MA 02142 USA; Open University, Milton Keynes, Buckinghamshire, MK7 6AA UK

**Keywords:** Lymphatic drainage, Basement membranes, Capillaries, Arteries, Amyloid-β, Nanoparticles neuroimmunology, Alzheimer’s disease, Virchow–Robin space, Drug delivery

## Abstract

In the absence of conventional lymphatics, drainage of interstitial fluid and solutes from the brain parenchyma to cervical lymph nodes is along basement membranes in the walls of cerebral capillaries and tunica media of arteries. Perivascular pathways are also involved in the entry of CSF into the brain by the convective influx/glymphatic system. The objective of this study is to differentiate the cerebral vascular basement membrane pathways by which fluid passes out of the brain from the pathway by which CSF enters the brain. Experiment 1: 0.5 µl of soluble biotinylated or fluorescent Aβ, or 1 µl 15 nm gold nanoparticles was injected into the mouse hippocampus and their distributions determined at 5 min by transmission electron microscopy. Aβ was distributed within the extracellular spaces of the hippocampus and within basement membranes of capillaries and tunica media of arteries. Nanoparticles did not enter capillary basement membranes from the extracellular spaces. Experiment 2: 2 µl of 15 nm nanoparticles were injected into mouse CSF. Within 5min, groups of nanoparticles were present in the pial-glial basement membrane on the outer aspect of cortical arteries between the investing layer of pia mater and the glia limitans. The results of this study and previous research suggest that cerebral vascular basement membranes form the pathways by which fluid passes into and out of the brain but that different basement membrane layers are involved. The significance of these findings for neuroimmunology, Alzheimer’s disease, drug delivery to the brain and the concept of the Virchow–Robin space are discussed.

## Introduction

There are two major extracellular, extravascular fluids associated with the brain, namely CSF in the ventricles and subarachnoid spaces and interstitial fluid (ISF) between cells within the CNS parenchyma. In most organs of the body, interstitial fluid, metabolites and antigen presenting cells drain to regional lymph nodes along well-defined lymphatic vessels. There are, however, no conventional lymphatic vessels in the CNS, but there are lymphatic drainage pathways by which CSF and ISF drain to regional lymph nodes.

CSF is produced mainly by the choroid plexuses and circulates through the cerebral ventricles and the subarachnoid spaces. Lymphatic drainage of CSF occurs through well-defined channels that traverse the cribriform plate of the ethmoid bone and carry fluid and tracers from the subarachnoid space to lymphatics in the nasal mucosa and to cervical lymph nodes [5]. In addition, there is lymphatic drainage of CSF along cranial and spinal nerve roots and into lymphatics in the dura mater [17]. In larger mammals, including humans, CSF also drains directly into the blood via arachnoid villi and granulations associated with venous sinuses.

Whereas there is a direct connection between the CSF and lymphatics in the nasal mucosa and dura mater, there is no direct link between ISF and conventional lymphatic vessels. Instead, lymphatic drainage of ISF occurs along basement membranes in the walls of cerebral capillaries and arteries at an estimated rate of 0.11–0.29 µl/min/g of brain [2, 23]. Although entry of ISF into lymph nodes from artery walls probably involves lymphatic vessels these have, as yet, not been defined. Evidence for lymphatic drainage along walls of cerebral blood vessels is derived from experimental studies and from observations in human cerebral amyloid angiopathy (CAA). When small volumes of soluble tracer are injected (0.5 μl injected over a period of 2 min) into the ISF of grey matter of the mouse striatum or hippocampus, tracer initially diffuses through the extracellular spaces of the brain but within 5 min has entered basement membranes in the walls of capillaries and cerebral arteries to drain out of the brain. If larger volumes are injected, tracer is not retained within the brain parenchyma and may pass into CSF in the ventricles [7]. Flow of ISF and tracers out of the brain along blood vessel walls is specifically along basement membranes between capillary endothelium and glia limitans and along basement membranes that surround smooth muscle cells in the tunica media of cerebral arteries in a three-dimensional network. Studies using radioactive human serum albumin have shown that tracers drain from the brain to cervical lymph nodes along the walls of leptomeningeal arteries and the internal carotid artery in the neck [23]. These experiments have also shown that only 5–10 % of tracer injected into the striatum leaks into the CSF, suggesting that the lymphatic drainage of ISF by the pericapillary and periarterial route is largely separate from the CSF [23]. One important role of intramural perivascular drainage along basement membranes lies in the transfer of soluble antigens from the CNS parenchyma to regional lymph nodes. The basement membrane pathways are too narrow to allow passage of antigen presenting cells and lymphocytes from the brain to regional lymph nodes and this may be a major factor for the immunological privilege of the brain [7, 8].

Another key role for intramural perivascular drainage along basement membranes is the elimination of solutes, such as soluble amyloid β (Aβ) from the brain. As arteries age, perivascular lymphatic drainage becomes less efficient and insoluble, fibrillary Aβ among other amyloids is deposited in the walls of cerebral capillaries and arteries as cerebral amyloid angiopathy (CAA) [24]. The distribution of Aβ in vascular basement membranes in the early stages of CAA in the human brain is exactly the same as the distribution of soluble tracers injected into the mouse brain, i.e. in capillary basement membranes and in basement membranes in the tunica media of arteries. In effect, Aβ produced by the brain acts as a natural tracer for lymphatic drainage in the human and mouse brain [13]. Basement membranes of arterial endothelium and basement membranes on the outer aspects of arteries are devoid of soluble tracer in the experimental animals and of Aβ in the early stages of human CAA. Thus, experimental data derived from mouse studies on lymphatic drainage of the brain are highly relevant for the human brain, CAA and Alzheimer’s disease.

The walls of cerebral arteries and capillaries are not only routes for the lymphatic drainage of ISF and solutes out of the brain, they are also routes by which CSF and tracers enter the brain from the subarachnoid space and mix with ISF. Injection of horseradish peroxidase [20] and a variety of fluorescent tracers into the CSF [15] results in their entry into the brain alongside cortical arteries and penetration of tracers into basement membranes surrounding capillaries.

This system has been termed convective influx [20] or the glymphatic [27] system as entry of tracers from the CSF into the ISF of the brain parenchyma involves aquaporin 4 in perivascular astrocytes.

The objective of the present study is to define, by transmission electron microscopy, the routes by which tracers pass into and out of the brain. We study the fine anatomy of the cerebral capillary wall and surrounding brain tissue to identify connections between the extracellular spaces and capillary basement membranes through which fluid and solutes may flow to enter the start of the perivascular lymphatic drainage pathway. Intramural perivascular drainage is used as the most suitable term and it is recognised that lymphatic drainage along the walls of arteries is actually along basement membranes within the tunica media rather than on the outside of the artery. In addition, the present study includes a comparison of the capacity of a soluble tracer, biotinylated Aβ, to pass from the extracellular spaces of cerebral grey matter into capillary basement membranes with the failure of 15 nm gold nanoparticles to pass by the same route.

To define the exact route by which tracers enter the brain from the CSF, we injected 15 nm gold nanoparticles into the CSF and traced their passage into the brain along basement membranes at the outer extremity of the artery wall between the coating of pia mater and the glia limitans. This entry pathway is distinct from the pathway along basement membranes surrounding smooth muscle cells in the tunica media by which drainage out of the brain occurs. The present study supports the hypothesis that vascular basement membranes are major routes for the transport of fluid and tracers into and out of the brain parenchyma.

## Materials and methods

### Intracerebral injections

All mice were kept on a standard 12-h light/dark cycle and allowed food and water ad libitum. All experiments were carried out in accordance with animal care guidelines stipulated by the Animal Care and Use Committee at the University of Southampton and the Home Office (PPL 30/3095). The anaesthetic used for all mice consisted of a combination of 10 mg/ml ketamine (Pfizer) with 1 mg/ml xylazine (Bayer), injected intraperitoneally 0.1 ml per 10 g body weight. The glass capillary micropipette routinely used for our intracerebral injections was purchased from Sigma UK and the tip was adjusted to a diameter of <50 μm using a Sutter P97 Flaming Brown Pipette puller. The exact volumes and concentrations for intracerebral injections are described below. Mice were culled at 5 min after withdrawal of the glass capillary through overdose with pentobarbital 200 mg/kg. Mice were intracardially perfused with 0.1 M piperazine-*N*,*N*′-bis(2-ethanesulfonic acid) buffer (PIPES, PH 7.2) followed by 3.4 % formaldehyde plus 3 % glutaraldehyde in 0.1 M PIPES buffer. Brains were removed through dissection from the skull, post-fixed overnight in fresh fixative and then sectioned into 200 μm thick coronal slices using a vibratome.

### Intraparenchymal injections of Aβ

To determine the ultrastructural location of soluble Aβ within the ISF as it moves from the parenchyma to the blood vessels, 10-week-old male wild-type C57Bl6 mice (*n* = 6) were micro-injected into the hippocampus with 0.5 μL biotinylated human Aβ40 of 100 μM concentration (AnaSpec, USA), using the injection technique described before [11]. Biotinylated Aβ was used to prevent any potential cross-reactivity between exogenous human Aβ and endogenous mouse Aβ. Three mice were injected only with phosphate buffered saline, serving as controls. The specificity of Aβ staining was confirmed by pre-incubating biotinylated Aβ injected tissue sections with avidin, to saturate the biotin binding sites bound the Aβ. Following this, the sections underwent 4G8 anti-Aβ immunohistochemistry using DAB as a chromagen (*n* = 3 mice) or immunofluorescence (*n* = 3) using streptavidin Alexa Fluor 546 (Invitrogen, UK).

To identify the clearance pathway of Aβ from the brains of naïve mice at the ultrastructural level, six free-floating sections of 100 μm thickness, from the hippocampus of mice injected with biotinylated human Aβ40 and from control mice injected with phosphate buffered saline were processed for enzyme-linked immunohistochemistry. Sections were washed 3 × 5 min in PBS before being treated with 70 % formic acid for 30 s. After washing for 3 × 5 min sections were treated with 3 % hydrogen peroxide for 30 min. The sections were washed a further 3 × 5 min in PBS. Following this, sections were treated for 1 h at room temperature with Vectastain™ ABC kit and developed using glucose oxidase enhancement with DAB as chromogen. Some sections were mounted onto frosted slides, dehydrated and coverslipped using DPX mounting medium. These sections were imaged using Nikon 80i brightfield microscope at 10× and 175× magnification and images were exported to Photoshop CS software, for orientation. Sections for TEM were transferred into 0.1 M phosphate buffer (PB), pH 7.2 for 3 × 5 min washes before being post-fixed with 1 % osmium tetroxide in 0.1 M PB, pH 7.2 for 1 h at room temperature. After further 3 × 5 min washes in PBS, the sections were dehydrated through an alcohol series (30 %–2 min, 50 %–2 min, 70 %–10 min, 90 %–10 min, 2 × 100 %–20 min). Sections were then treated with acetonitrile for 10 min before being left in a 50:50 mixture of acetonitrile and TAAB resin (TAAB laboratories, UK) for 12 h. Following resin infiltration, sections were placed into fresh TAAB resin for 6 h before being embedded in fresh TAAB resin and were polymerised at 60 °C for 48 h. Polymerised resin blocks were sectioned using a Reichurt Ultracut E ultramicrotome (Reichurt, Germany). Semi-thin sections (0.5 μm thick) were cut and stained with toluidine blue (Agar Scientific, UK). These sections were analysed using a Nikon 80i brightfield microscope at 10× and 175× magnification to assess the quality of the tissue. Regions with good ultrastructural preservation were further microdissected. Ultra-thin sections (90 nm thick) were cut from the microdissected block using a diatome diamond knife and floated on to TEM grids for visualisation. In total, 36 sections were analysed by TEM and Aβ was observed within 5–10 capillaries per section. Examination through the section covered every grid square from top right to bottom left. Analysis was performed using a Hitachi H7000 transmission electron microscope. Images were captured using iTEM software (Universal TEM Imaging Platform, Soft Imaging System, Münster, Germany) operating a MegaView III digital camera (Soft Imaging System, Münster, Germany).

### Immunofluorescence for the localisation of Aβ

For double labelling immunohistochemistry, tissue sections were incubated overnight with anti-α smooth muscle actin (1:500, Sigma-Aldrich, Dorset, UK) and anti-laminin (1:500, Sigma-Aldrich, Dorset, UK) and developed with Alexa Fluor-conjugated secondary antibodies (Invitrogen, Paisley, UK). Photomicrographs of triple labelling immunohistochemistry were captured from tissue sections at least 100 μm away from the injection site using a Leica SP5 confocal laser scanning microscope (Milton Keys, UK) and exported to Photoshop CS software.

### Synthesis of citrate coated spherical gold nanoparticles of 15 nm diameter

Sodium citrate stabilized spherical gold nanoparticles (NPs) were prepared using the Turkevich method [18]. Briefly, 2.5 ml of 19.5 mM trisodium citrate was brought to boil and quickly mixed with a boiled solution of sodium tetrachloroaurate (III) dehydrate (0.5 mM, 25 ml) whilst stirring vigorously (the solution changes colour from pale yellow to colourless, then to purple and finally to deep red indicating the formation of 15 nm spherical gold NPs). After 5 min the solution was cooled to room temperature and filtered using a 0.45 μm syringe filter. The citrate coated NPs were then capped with bis-(*p*-sulfonatophenyl) phenylphosphine dihydrate dipotassium (BSPP) by mixing 27.5 ml of gold NPs with 10 mg of BSPP and stirring overnight, precipitated using 50 mg of sodium chloride and purified by centrifugation at 5000 rpm for 5 min at 20 °C. The resulting nanoparticles were re-dispersed by sonication in 100 μl of Milli-Q water and stored at 4 °C. The size of the gold nanoparticles was confirmed by TEM using 3.05 mm diameter Carbon coated 400 mesh Copper grids and UV–visible spectroscopy. Gold NP concentration was estimated using Beer–Lambert law and then diluted in Milli-Q water to the desired concentration.

### Surface functionalization of 15 nm gold nanoparticles

Citrate coated spherical NPs of 15 nm diameter were coated with Polyethylene glycol (PEG) compounds containing either a positive (SH-PEG-COOH), neutral (SH-PEG-OCH_3_) or negative (SH-PEG-NH_2_) function group. For each function group, NPs (5 nm, 10 ml) were mixed with the PEG compound (5 mg, 200 μl) whilst continually stirring, incubated for 2 h on a shaker (500 rpm) and left overnight at 4 °C. Functionalised gold NPs were then purified by centrifugation (3 × 16,400 rpm, 15 min, 10 °C) and re-dispersed by sonication in 100 μl of Milli-Q water.

### Intraparenchymal injections of 15 nm Gold nanoparticles

10-week-old male mice were injected stereotactically with 1 μl of either positive, negative or neutrally charged 15 nm gold nanoparticles (*n* = 3/group) into the left hippocampus over 5 min, at a rate of 0.2 μl/min (coordinates from Bregma: AP = −1.9 mm; ML = 1.5 mm and DV = −1.3 mm). Injection pipettes were left in situ for 2 min to prevent reflux and mice were killed 5-min postinjection.

Coronal slices at 200, 400, 600 and 800 μm anterior and posterior to the hippocampi injection site were prepared for examination by TEM. For each 100 μm slice four 1 mm^2^ samples were microdissected from the left hippocampus and processed for TEM (32 × samples per mouse, 96 × samples per group). TEM was performed by methodically scanning each sample from top right to bottom left with areas containing nanoparticles digitally photographed.

### Injections of 15 nm gold nanoparticles into Cisterna Magna

Anaesthetised 10-week-old male mice (*n* = 3) were placed prone on a stereotaxic instrument and secured with head adaptors. A sagittal incision of the skin was made inferior to the occiput, the subcutaneous tissue and biventer cervicis and rectus capitis dorsalis major muscles were bluntly separated to expose the posterior atlanto-occipital membrane that was incised to reveal the dura mater of the cisterna magna. The position of the mouse was then readjusted so that the head formed a 135° angle with the body. The exposed dura mater was then pierced with the injection pipette lateral to the arteria dorsalis spinalis and 2 μl of 15 nm negative gold nanoparticles was injected over 2.5 min, at a rate of 0.8 μl/min. Injection pipettes were left in situ for 2 min to prevent reflux and mice were killed 5 min postinjection. Perfusion-fixation, removal and sectioning of brains were performed as described above. The parietal cortex and corpus callosum were microdissected and processed for TEM according to methods developed by us and described above and in previous studies [22].

## Results

### Connections between extracellular spaces in grey matter of the brain and pericapillary basement membranes

**Fig. 1:**
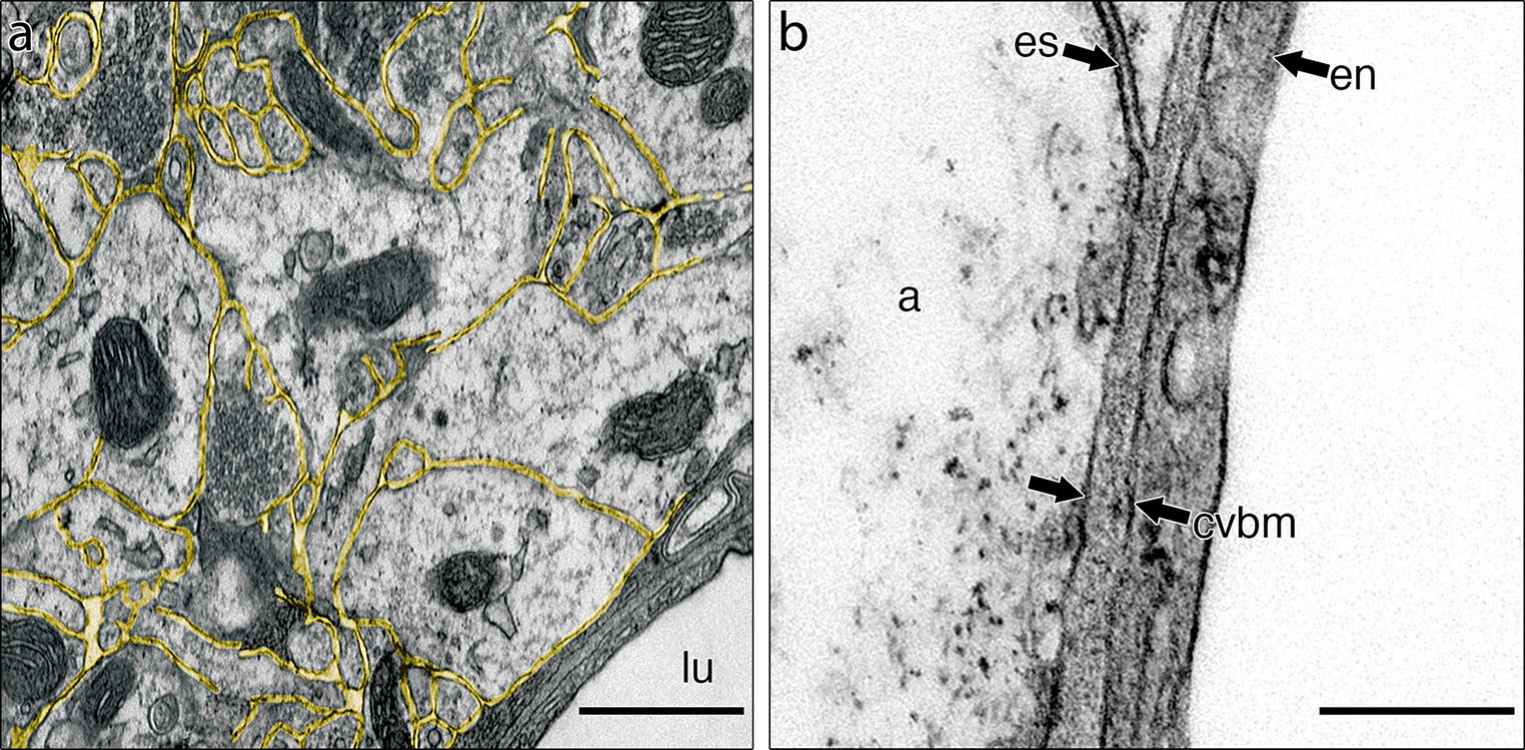
Extracellular space in grey matter of the hippocampus and its connection to capillary basement membrane. **a** Grey matter with the extracellular space outlined in *yellow*. There is a capillary in the bottom left-hand corner. Capillary lumen (*lu*). **b** High-magnification view of the junction of the extracellular space (*es*) with the capillary basement membrane (*cvbm*) at the level of the astrocyte membrane. Endothelial cell of the capillary (*en*); astrocyte of the glia limitans (**a**). *Scale bars* **a** 500 nm; **b** 250 nm

Electron microscope preparations were used to examine the relationship between the extracellular spaces in the mouse hippocampus and basement membranes surrounding capillaries. Figure 1a shows the narrow and tortuous extracellular spaces in the brain parenchyma outlined in yellow and how they abut on to the walls of capillaries. At higher magnification in Fig. 1b, the interface of the extracellular space between astrocyte processes and the basement membrane of the capillary wall can be demonstrated. The basement membrane around capillaries is formed from two elements derived on one side from the astrocytes of the glia limitans and on the other side from capillary endothelium; it is in direct focal contact with the extracellular space of the brain.

### Distribution of biotinylated Aβ injected into the hippocampus

From previous experiments using fluorescent tracers [7] it was estimated that 5 min after injection would be the optimal time to examine the relationship between biotinylated Aβ in the extracellular spaces of the brain and the presence of biotinylated Aβ in capillary basement membranes. At later times, fluorescent tracer is reduced in amount in the extracellular spaces and this is accompanied by reduced presence of tracer in capillary basement membranes.

**Fig. 2:**
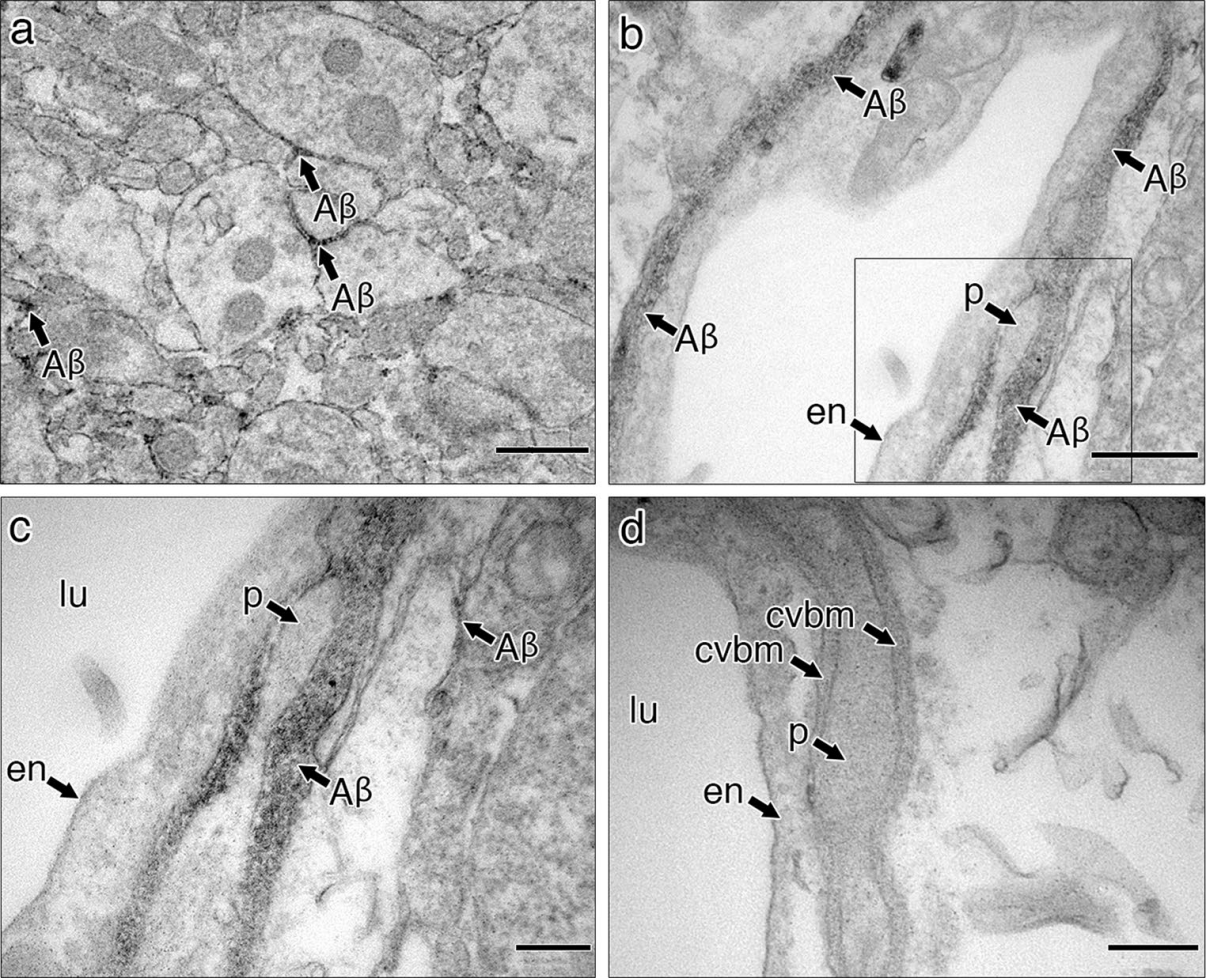
Biotinylated Aβ identified in the extracellular space of hippocampal grey matter and in a capillary basement membrane. **a** Hippocampal neuropil showing darkly stained biotinylated Aβ in the extracellular spaces (*arrows* labelled Aβ); **b** capillary in the hippocampus showing darkly stained biotinylated Aβ in the basement membrane; **c** high-magnification view of the area within the square in Fig. 2b showing darkly stained biotinylated Aβ in the extracellular space (*right*) and throughout the capillary basement membrane (*left*) that encapsulates a pericyte (*p*); **d** capillary from a control hippocampus, into which no biotinylated Aβ has been injected. The basement membrane (*cvbm*) is negative for Aβ staining. *en* endothelium, *lu* lumen, *p* pericyte, *cvbm* cerebrovascular basement membrane. *Scale bars* **a** 500 nm; **b**–**d** 200 nm

At 5 min after injection into the hippocampus, densely staining biotinylated Aβ is seen in the narrow extracellular spaces between neuronal and glial processes (Fig. 2a). Examination of capillary walls at the same time interval reveals the presence of dark granular staining for biotinylated Aβ within the capillary basement membrane (Fig. 2b). At higher magnification (Fig. 2c), staining for Aβ extends throughout the width of the basement membrane and on either side of a pericapillary pericyte. Images were compared with basement membranes from capillaries of animals that had received injections of phosphate buffered saline and no injection of biotinylated Aβ (Fig. 2d); basement membranes in non-injected animals show no granular dense staining for Aβ.

### Aβ in basement membranes of the tunica media of leptomeningeal arteries 5 min after injection of fluorescent Aβ into the hippocampus

**Fig. 3:**
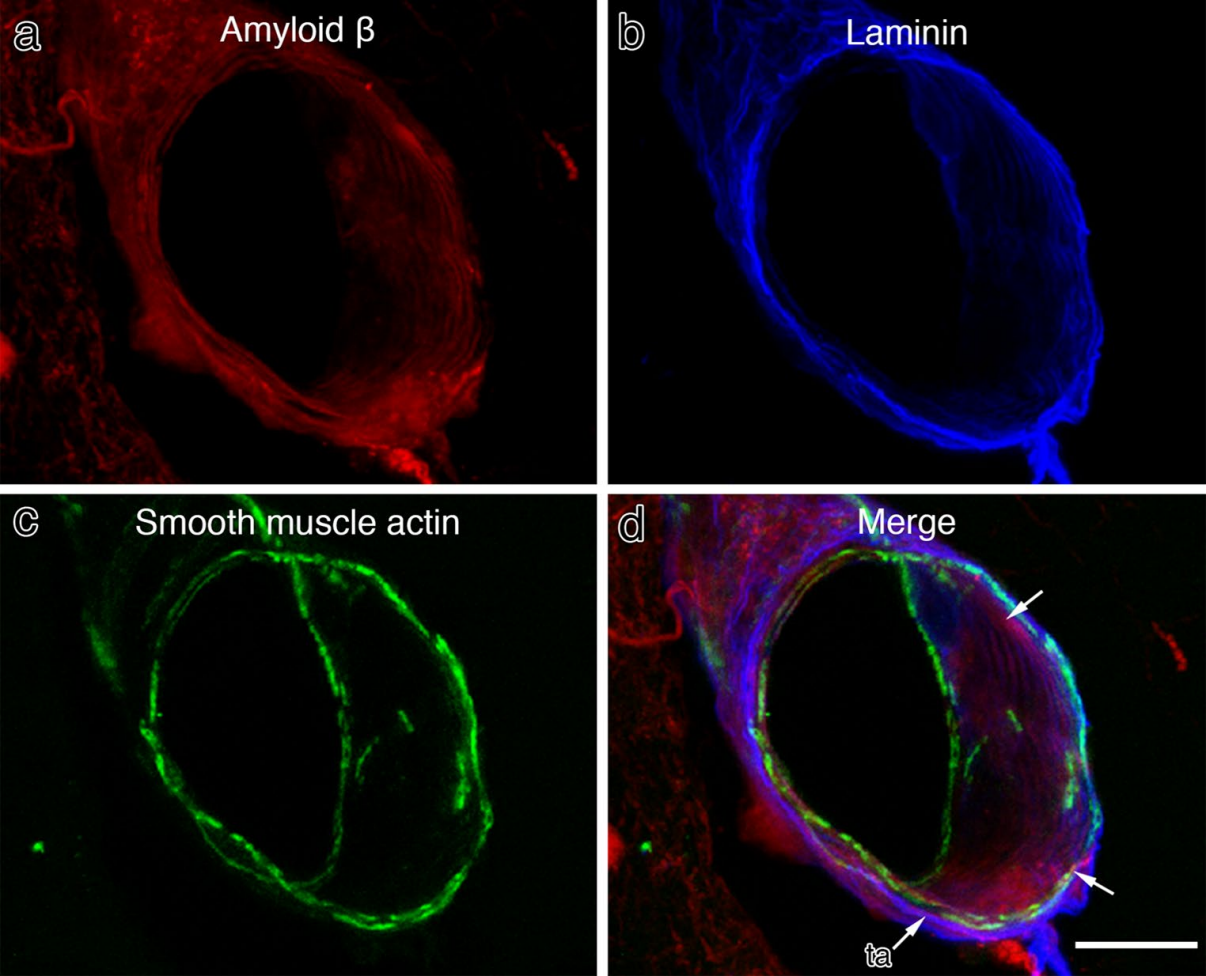
Immunocytochemistry/confocal images showing that 5 min after the injection of soluble Aβ into the hippocampus, Aβ is associated with basement membrane proteins within the wall of an artery in the hippocampal fissure. **a** Aβ (*red*) in the wall of a leptomeningeal artery; **b** laminin (*blue*); **c** a layer of smooth muscle cells (*green*) in the artery wall that is most clearly seen when the vessel is cut in cross section; **d** a merged image showing co-localisation of Aβ and laminin (*purple*–see *arrows*) associated with the smooth muscle cells. The outermost layer of the artery, the tunica adventitia (*ta*), is stained only for laminin (*blue*) and not for Aβ. SP5 Leica confocal image. *Scale bar* 50 μm

To demonstrate that soluble Aβ injected into the hippocampus reaches leptomeningeal arteries 5 min after injection, sections of brain were examined by immunocytochemistry and confocal microscopy. Figure 3 shows a series of confocal images that depict staining for Aβ, basement membrane laminin and smooth muscle actin. The leptomeningeal artery in Fig. 3 is seen as a cylinder viewed obliquely and end-on. Aβ (Fig. 3a) has a similar pattern of staining in the vessel wall to laminin (Fig. 3b) but not exactly the same. Differences can be detected in the merged image (Fig. 3d); although Aβ merges with laminin throughout the length of the section of the vessel wall, the tunica adventitia on the outer aspect of the artery stains for laminin but is devoid of Aβ. Figure 3c shows a thin but complete circumferential coat of smooth muscle cells in the artery wall that is most strongly visible where the artery is cut in transverse section. The completeness of the smooth muscle coat allows arteries to be distinguished from veins that have only occasional smooth muscle cells in their walls. Basement membrane staining for laminin and Aβ is associated with the smooth muscle cell layer (Fig. 3d). These images support previously published data [7] showing fluorescent tracers within the basement membranes of the tunica media of cerebral arteries. No tracer was observed in association with veins.

### Distribution of 15 nm gold nanoparticles in the hippocampus 5 min after injection

**Fig. 4:**
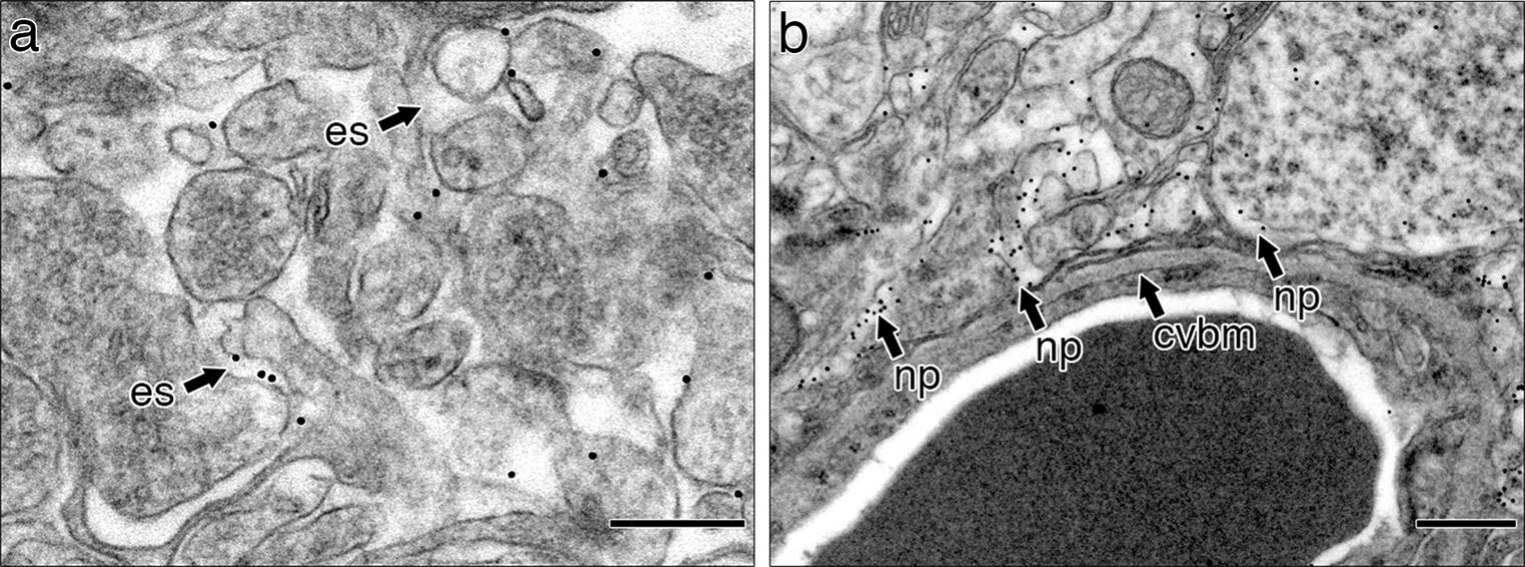
Nanoparticles are distributed in the extracellular spaces but do not enter the capillary basement membrane. **a** Densely stained punctate nanoparticles are seen in the extracellular spaces (*es*) of the brain and within cell processes of neurons and possibly of astrocytes. **b** Neuropil and capillary showing nanoparticles (*np*) in the extracellular spaces of the neuropil and occasionally in cell processes but no nanoparticles are located in the capillary basement membrane (*cvbm*). *Scale bars* **a** 300 nm; **b** 500 nm

To test the capacity of the pericapillary route for lymphatic drainage, 15 nm gold nanoparticles were injected into the grey matter of the hippocampus and specimens were examined 5 min after injection. In this way the results could be compared with the injections of biotinylated Aβ. Gold nanoparticles were distributed through the extracellular spaces of the hippocampal parenchyma and some were taken up by neuronal and possibly by astrocyte processes (Fig. 4a). However, no nanoparticles were identified within capillary basement membranes (Fig. 4b) although they were in the pericapillary extracellular spaces and within adjacent neuronal processes. These results suggest that although 15 nm nanoparticles are widely distributed through the extracellular spaces they do not enter basement membranes of the perivascular lymphatic drainage pathway.

### Distribution in the brain of 15 nm nanoparticles injected into the CSF

We have shown that soluble biotinylated Aβ injected into the extracellular spaces of the hippocampus readily enters basement membranes around capillaries that form the initial part of the lymphatic drainage pathway of the brain. 15 nm nanoparticles, on the other hand, do not enter capillary basement membranes from the extracellular spaces of the brain parenchyma. We therefore tested the capacity of the entry route for CSF into the brain by injecting 15 nm nanoparticles into the CSF. Here we show that 15 nm nanoparticles do enter the brain from the CSF but that the route of entry is along basement membranes on the outer aspect of arteries as they enter the cerebral cortex. No nanoparticles were observed around veins and no nanoparticles entered the extracellular spaces of the brain parenchyma around arteries.

**Fig. 5:**
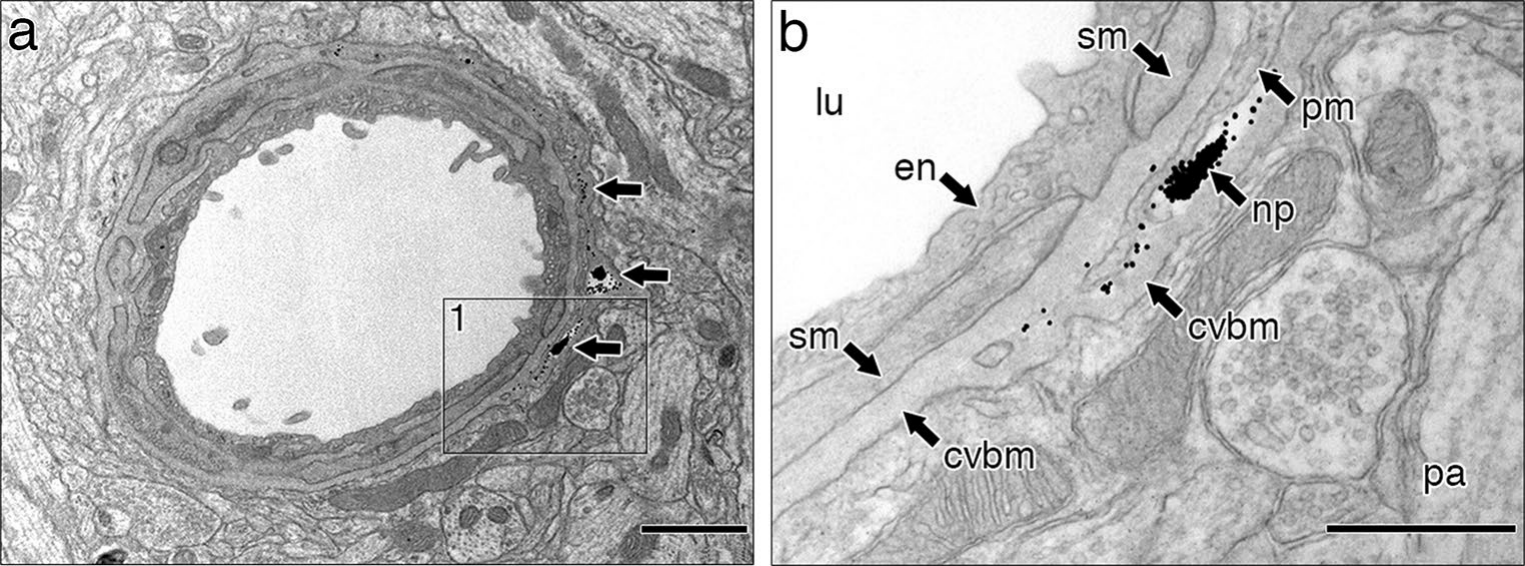
Distribution in the brain of 15 nm nanoparticles injected into the CSF. Groups of 15 nm nanoparticles are present in the pial-glial basement membrane of a cortical artery 5 min after injection into the CSF of the cisterna magna. **a** Low-power transmission electronmicrograph of an artery near the surface of the cerebral cortex. Note that cells and basement membranes form compact layers and there is no Virchow–Robin space. Dense nanoparticles form lines or groups in the pial-glial basement membrane between the pia mater and the glia limitans around nearly half the circumference of the artery but no nanoparticles are seen in basement membranes between smooth muscle cells. **b** Higher magnification of the square labelled 1 in **a**. Layers of the artery wall can be identified moving outwards from the lumen (*lu*) through the endothelium (*en*), smooth muscle coat (*sm*) and a layer of pia mater (*pm*) to the glia limitans of the brain parenchyma (*pa*). Dense groups and single nanoparticles (*np*) are located mainly in the pial-glial basement membrane (*cvbm*) between the pia mater (*pm*) and the adjacent glia limitans. *Scale bars* **a** 1 μm; **b** 500 nm

Figure 5 shows an artery near the surface of the mouse cerebral cortex with a compact wall and no perivascular space. An area of wall in the square box in Fig. 5a is shown at higher magnification in Fig. 5b. A group of 15 nm gold nanoparticles with some single nanoparticles is present mainly in the basement membrane on the outer aspect of the artery between the layer of leptomeningeal pia mater and the astrocyte layer of glia limitans of the brain (Fig. 5b). This layer of basement membrane is shared by pia mater and glia limitans (the pial-glial basement membrane) and is distinct from the layers of basement membrane surrounding smooth muscle cells in the tunica media that act as the pathway for drainage of fluid and solutes out of the brain. Figure 5a, b also shows that there is no space between layers of the artery wall and the glia limitans.

## Discussion

The major purpose of the present study was to gather further evidence for the hypothesis that vascular basement membranes form the pathways for passage of fluid into and out of the brain. We first showed that when biotinylated Aβ is injected in small volumes into the hippocampal grey matter, it spreads through the extracellular spaces and enters basement membranes of capillaries within 5 min of injection. Electron microscope images show continuity between the extracellular spaces and capillary basement membranes. Using fluorescent Aβ, we showed by confocal microscopy that it was also present in the basement membrane associated with smooth muscle cells in leptomeningeal arteries within 5 min. When injected into the hippocampus, 15 nm gold nanoparticles spread through the extracellular spaces but they did not enter the basement membranes of capillaries. This suggests that there is a limit on size, rigidity and perhaps on charge of material that will enter capillary basement membranes to drain out of the brain along the intramural perivascular drainage pathway.

Our second series of experiments showed that when 15 nm gold nanoparticles were injected into the CSF in the cisterna magna, they rapidly entered the brain alongside artery walls. Within 5 min, nanoparticles were present in the pial-glial basement membranes on the outer aspects of arteries penetrating the cerebral cortex but did not enter the extracellular spaces of the brain parenchyma. The pial-glial basement membrane pathway for the entry of tracers from the CSF into the brain is separate from the pathway for intramural perivascular drainage of fluid and solutes out of the brain that is situated within the smooth muscle cell basement membranes in the tunica media of arteries [7]. The drainage of solutes along the basement membranes of capillaries and arteries is towards the large leptomeningeal arteries most likely to the deep cervical lymphatics. From the striatum, solutes may appear to drain also into the ventricles, probably an underlying mechanism for the elimination of periventricular oedema that is observed in some neurological conditions or after head trauma [4].

**Fig. 6:**
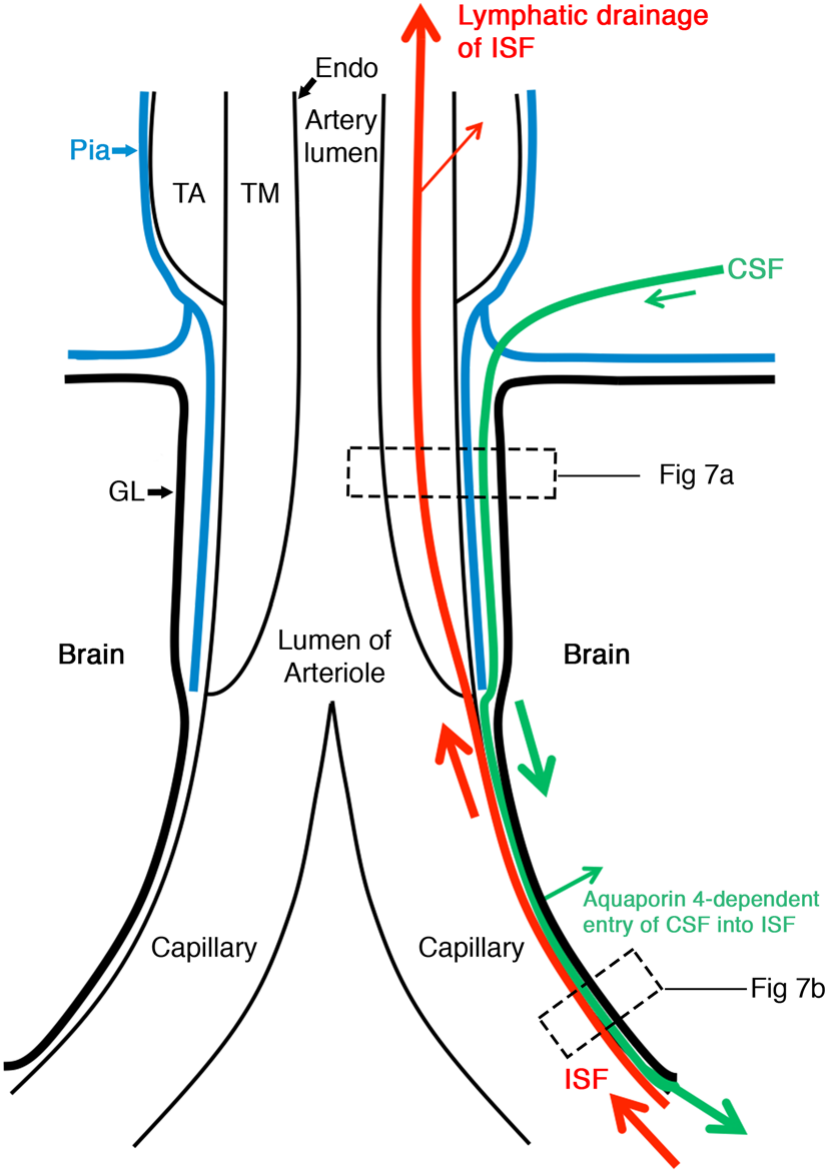
Schematic representation of the lymphatic drainage and convective influx/glymphatic systems of the brain. An artery enters the brain from the subarachnoid space and an arteriole divides into capillaries. At the top of the figure, the artery is lined by endothelium (*Endo*), and coated by the tunica media (*TM*) composed of smooth muscle cells and by the outermost tunica adventitia (*TA*) composed of connective tissue. As it enters the brain, the artery loses the tunica adventitia but is still coated by a layer of pia-arachnoid (*Pia*) that intervenes between the artery and the glia limitans (*GL*) of the brain. As the arteriole divides into capillaries, the tunica media and the layer of pia mater are lost. Thus, at the level of the capillary, the glia limitans is in direct contact with the wall of the capillary. On the *right-hand side* of the diagram, the *red arrows* indicate the intramural perivascular lymphatic drainage pathway by which interstitial fluid (*ISF*) and solutes pass out of the brain along basement membranes in the walls of capillaries and along basement membranes surrounding smooth muscle cells in the tunica media of arterioles and arteries [7]. Tracers in the CSF enter the brain along the pial-glial basement membrane between the pia mater and the glia limitans (indicated by a *green arrow*) and enter the brain parenchyma and interstitial fluid by an aquaporin 4-dependent mechanism, which is the convective influx/glymphatic pathway

**Fig. 7:**
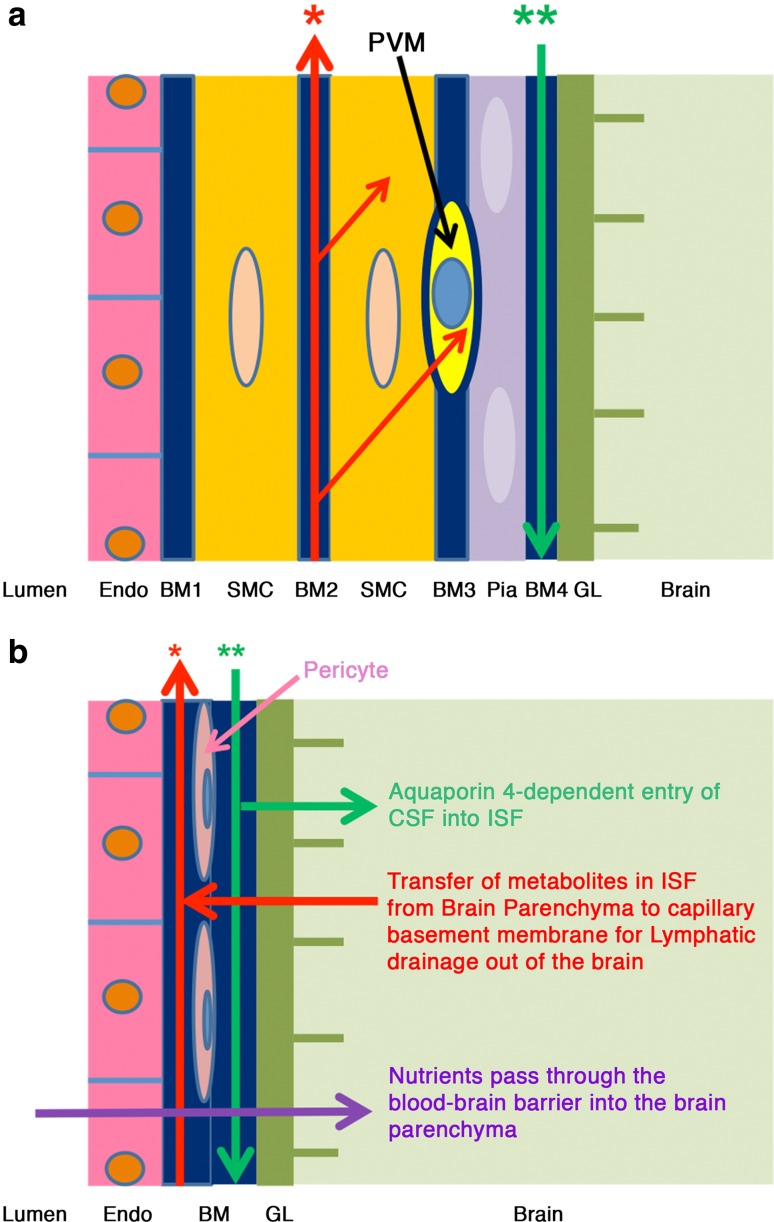
Expanded view of the wall of a cerebral artery near the surface of the cerebral cortex. **a** The lymphatic drainage pathway for interstitial fluid (*ISF*) and solutes is specifically along basement membranes surrounding smooth muscle cells in the tunica media (*red arrow* *). Tracer studies have shown that solutes are taken up by smooth muscle cells and by perivascular macrophages (*PVM*) (*thin red arrow*s). The convective influx/glymphatic pathway by which CSF enters the brain is along the pial-glial basement membrane (BM4–*green arrow* **) between the layer of pia mater and the glia limitans of the brain parenchyma. Abbreviations for structure of the artery wall: *Endo* endothelium, *BM* basement membrane, *SMC* smooth muscle cells, *GL* glia limitans, *BM1* basement membrane formed by the endothelium and adjacent smooth muscle cells, *BM2* basement membrane formed by adjacent smooth muscle cells; this is the pathway for the lymphatic drainage of interstitial fluid and solutes from the brain along the tunica media, *BM3* basement membrane formed by the outer smooth muscle cells and pia mater cells coating the artery, *BM4* basement membrane formed by the pia mater and astrocytes of the glia limitans. It is along BM4 that CSF and nanoparticles enter the brain in the convective influx/glymphatic system. **b** Expanded view of the wall of a cerebral capillary and surrounding glia limitans. The endothelium has a basement membrane that is formed partly by endothelial cells and partly by astrocytes of the glia limitans. Solutes, such as amyloid β (Aβ) in the interstitial fluid pass from the extracellular spaces of the brain into the endothelial-glial basement membrane and drain along the intramural perivascular drainage pathway (*red arrow*). Tracers injected into the CSF reach the capillary basement membranes via the convective influx/glymphatic system and their entry into the interstitial fluid is dependent upon aquaporin 4 (*green arrows*). Nutrients from the blood through the blood–brain barrier pass into the brain through the endothelium, the basement membrane and the glia limitans (*purple arrow*). Pericytes are surrounded by basement membrane and lie between the endothelium (*Endo*) and the basement membrane (*BM*) of the glia limitans (*GL*). Although the *red* and *green arrows* are shown in separate parts of the basement membrane, a single capillary basement membrane appears to share traffic of fluid into and out of the brain

The findings in this study have been combined with previous observations and summarised in Figs. 6 and 7. ISF and soluble tracers flow out of the brain along basement membranes initially in the walls of capillaries and then along basement membranes surrounding smooth muscle cells in the tunica media of arteries (shown as a continuous red line in Fig. 6). Tracers from the CSF, on the other hand, enter the brain along basement membranes on the outside of arteries between the pia mater covering of the artery and the glia limitans (Fig. 6). Previous studies have shown that when particulate tracers such as Indian ink are injected into the cisterna magna they pass into basal cisterns of the subarachnoid space and spread along narrow channels either side of major cerebral arteries over the lateral surfaces of the hemisphere [17]. From this compartment of the subarachnoid space, nanoparticles appear to pass through the pia mater on the surface of the brain to enter the pial-glial basement membranes on the outer aspects of penetrating arteries as shown in Fig. 6. A recent study demonstrated that soluble tracers penetrated the parenchyma after injection into cisterna magna, with 3 kDa tracers reaching 64 μm cortical depth [4]. The permeability characteristics of the mouse pia mater have not been fully defined and require further investigation in future studies.

The separation between entry and exit routes is emphasised in Fig. 7a. The entry route is depicted by a green arrow with two asterisks in the pial-glial basement membrane, whereas the route for the lymphatic outflow of ISF and tracers is shown by the red arrow with one asterisk associated with smooth muscle cells in the tunica media. Uptake of tracer by smooth muscle cells in the artery wall suggests that solutes passing out of the brain by this pathway may have a signalling function for vascular activity. Similarly, the uptake of tracers by perivascular macrophages may serve an immunological function or act as a mechanism for elimination of metabolites as they pass along the lymphatic drainage pathways [12].

Figure 7b is a detailed view of a capillary wall and the surrounding glia limitans. Results of the present study suggest that soluble tracers injected into the parenchyma of the hippocampus enter capillary basement membranes and then flow out of the brain along arterial basement membranes. Rennels showed in 1985 that when the soluble tracer horseradish peroxidase is injected into the CSF it penetrates the brain along the outer aspects of artery walls and reaches capillary basement membranes [20]. Aquaporin 4 is involved in the transfer of tracers from perivascular basement membranes into the ISF of the brain parenchyma [15] but the mechanisms of transfer of ISF and solutes from the brain parenchyma into capillary basement membranes are unclear. There also appears to be traffic of nutrients through capillary basement membranes as they pass into the brain via the blood–brain barrier [1] making the system even more complex (Fig. 7b).

### Basement membranes as routes for transport of fluid and solutes in and out of the brain

The present study demonstrates that the anatomical compartments for bulk flow of fluid into and out of the brain are along basement membranes. All the vascular basement membranes involved in this process appear to be composed of two elements. Capillary basement membranes are formed by the fusion of elements from endothelium and from glia limitans. Basement membranes in the tunica media are formed by fusion of elements from adjacent smooth muscle cells. The pial-glial basement membrane that acts as a pathway for the entry of tracers from the CSF is partly contributed by the pia mater and partly by the glia limitans. Ultrastructural studies have identified three layers in such basement membranes, two outer laminae rarae and a central lamina densa at the site where the two elements of the basement membrane fuse. Although in the present study, Aβ filled the whole width of the capillary basement membrane, there is some indication that the lamina densa may be the route for transport of solutes along basement membranes between vascular smooth muscle cells. Fibrils of insoluble Aβ first precipitate in the lamina densa in the very early stages of cerebral amyloid angiopathy (CAA) [26]. Subsequently deposits of Aβ expand and separate the two elements of adjacent smooth muscle basement membrane [16]. Similar separation of the two elements of the glio-pial basement membrane occurs when particles are injected into the brain and spread between walls of arteries and the surrounding glia limitans [29].

### Significance for neuroimmunology, Alzheimer’s disease, drug delivery and the concept of the Virchow–Robin space

#### Neuroimmunology

Drainage of solutes but not nanoparticles from the extracellular spaces of the brain into capillary basement membranes is the start of the rapid and direct lymphatic drainage pathway to cervical lymph nodes. This pathway continues from capillaries along basement membranes in the tunica media of cerebral and leptomeningeal arteries; it allows passage of solutes at least up to 150 kDa [3] but not the passage of 15 nm nanoparticles as shown in the present study. Immune complexes consisting of antigen, antibody and compliment becoming entrapped in the basement membrane drainage pathway in arteries and temporarily impair perivascular lymphatic drainage [9]. These observations suggest that there is a size limit on material passing directly from the brain parenchyma to lymph nodes. It is likely that the basement membrane pathways are not large enough to allow passage of antigen presenting cells or lymphocytes directly from the brain to lymph nodes. This has direct implications for neuroimmunology and the failure of direct transport of antigen presenting cells and lymphocytes from the brain to regional lymph nodes may play a role in immunological privilege in the brain [19].

#### Alzheimer’s disease

Perivascular lymphatic drainage pathways are routes for the elimination of Aβ from the brain parenchyma as shown by injection studies in the mouse. As elimination of Aβ fails with ageing of cerebral blood vessels, Aβ accumulates in the walls of arteries as cerebral amyloid angiopathy [16]. Age-related failure of elimination of Aβ probably reflects failure of elimination of other metabolites and is associated with a rise in the level of soluble Aβ in the brain that in itself reflects failure of homoeostasis of the extracellular environment [25]. Failure of elimination of Aβ along capillary and arterial basement membranes with age could be the trigger for the amyloid cascade that drives the deposition of insoluble Aβ in plaques in the brain and furthermore may promote the propagation of tau pathology and neuro fibrillary tangles in the brain in Alzheimer’s disease [25].

#### Drug delivery

Convection-enhanced delivery (CED) is a strategy for injecting drugs into the brain parenchyma to bypass the bloodbrain barrier. This technique has shown significant potential in clinical trials for the treatment of malignant gliomas and Parkinson’s disease [6, 10]. The present study emphasises the anatomical basis for this technique. Solutes up to 150 kDa drain rapidly out of the brain along basement membranes of cerebral capillaries and cerebral arteries following simple injection or CED [3, 7]. Solutes of 70 and 150 kDa show some delay drainage [3]. Particulate matters such as 15–20 nm nanoparticles, 0.2 μm fluorospheres and Indian ink do not enter the basement membranes of the lymphatic drainage pathway but they do track along the pial-glial basement membrane and separate the glia limitans from the surface of arteries [7, 29]. Indian ink injected into the rat brain remains in situ around arteries for up to 2 years having been ingested by perivascular macrophages [29]. Uptake of particles at this site by perivascular macrophages is not as rapid as the uptake of solutes draining along the intramural perivascular pathways within the tunica media of arteries [3]. The compartment that is expanded by the injection of particulate matter into the brain parenchyma is the same pial-glial compartment along which nanoparticles enter the brain from the CSF as shown in the present study.

### The concept of the Virchow–Robin space

The term “Virchow–Robin space” is widely used to denote dilated perivascular spaces particularly in the basal ganglia and white matter of the brain. It has also long been assumed that there is a physically empty space between the walls of arteries penetrating the cerebral cortex and the surrounding brain tissue [5]. However, ultrastructural studies by scanning and transmission electron microscopy have demonstrated that first a layer of pia mater separates the subarachnoid space from the subpial space and perivascular compartments [14] and secondly, there is a compact series of layers of cell processes and basement membrane between the walls of arteries and the surrounding brain tissue and no space as shown in the mouse in the present study (Figs. 5, 6) and in the human brain [28]. There is no empty “Virchow–Robin space” between the artery wall and the glia limitans. Apparent spaces are commonly seen in paraffin sections of brain tissue but the space appears to be within brain tissue and artefactual as often there are astrocyte processes crossing the space [21]. Injection of particulate matter into the brain does separate elements of the pial-glial basement membrane to form a perivascular space [7, 29].

## Conclusions

This study has determined the role of cerebral vascular basement membranes as pathways for the passage of fluid and tracers into and out of the brain. Soluble tracers such as Aβ enter capillary basement membranes from the extracellular spaces and drain out of the brain along the basement membranes of cerebral capillaries and arteries as part of the rapid and direct lymphatic drainage of the brain parenchyma. This pathway does not allow drainage of nanoparticles or of antigen presenting cells to regional lymph nodes; it also fails as arteries age and is further blocked by the deposition of amyloid in cerebral amyloid angiopathy. Lymphatic drainage pathways play a significant role in neuroimmunology and are an important factor in maintaining immunological privilege. Failure of elimination of Aβ along perivascular drainage pathways is a significant factor in the development of Alzheimer’s disease. A different periarterial pathway allows CSF and nanoparticles to penetrate the brain from the CSF as part of the convective influx/glymphatic system; this pathway is the pial-glial basement membrane that can be expanded by particulate matter. Questions still remain regarding the dynamics and physiology of basement membrane pathways for transport of fluid into and out of the brain, not least is the question of how the capillary basement membrane appears to allow passage of fluid and tracers in more than one direction within the same structure.
